# Effect of glucocorticoids on aquaporin-1 in guinea pigs with otitis media with effusion

**DOI:** 10.3892/etm.2013.1036

**Published:** 2013-04-02

**Authors:** CHENJIE YU, XINYAN CUI, FENG CHEN, JUN YANG, XIAOYUN QIAN, XIA GAO

**Affiliations:** 1 Department of Otolaryngology, Head and Neck Surgery, Drum Tower Hospital, Nanjing University School of Medicine, Nanjing, Jiangsu 210008;; 2 Department of Otolaryngology, The First Affiliated Hospital, Nanjing Medical University, Nanjing, Jiangsu 210029;; 3 Department of Pathology, Drum Tower Hospital, Nanjing University School of Medicine, Nanjing, Jiangsu 210008, P.R. China

**Keywords:** glucocorticoid, aquaporin-1, guinea pig, pathophysiology

## Abstract

The aim of this study was to explore the pathological changes in water homeostasis and the effects of glucocorticoids on aquaporin-1 (AQP1) in guinea pigs with otitis media with effusion (OME). Immunohistochemistry and western blotting were used to detect AQP1 in the bullae of OME models, which were induced by reversible Eustachian tube (ET) obstruction. Animals in the dexamethasone (dexa) group received dexa via intraperitoneal injection for 7 days and the pathological changes and expression patterns of AQP1 were compared with those in the OME group. In this study, 22 guinea pigs exhibited effusion 3–7 days after surgery, of which two were sacrificed. Six (60%) animals in the OME group and 9 (90%) in the dexa group presented no sign of effusion on postoperative day 14. AQP1 was detected as an 28-kDa protein in the two groups. Immunohistochemical analysis revealed that AQP1 was expressed in subepithelial fibroblasts and capillary endothelial cells. Western blot analysis revealed that the levels of AQP1 protein were markedly higher in the dexa group compared with the OME group. In conclusion, our study emphasized the significance of AQP1 in the pathophysiology of OME and suggests that glucocorticoids may regulate water homeostasis via an AQP1-regulated pathway.

## Introduction

Otitis media with effusion (OME) is a common disorder characterized by inflammation of the middle ear (ME), with accumulation of fluid. OME is a worldwide problem that leads to conductive hearing loss in children, resulting in developmental problems in speech, language and the acquisition of social skills (1,2). Previous studies have suggested that intrinsic or acquired Eustachian tube (ET) obstruction, nasal allergies and/or bacterial infection of the ME may be involved in the development and persistence of ME mucosal inflammation; however, the exact etiology of OME remains unclear (3–5).

Aquaporins (AQPs) are integral membrane proteins that serve as channels for the transfer of water and, in certain cases, small solutes across the membrane (6). AQPs play a significant role in water balance (local homeostasis) and deficits in AQP expression and/or function have been associated with several diseases of dysfunctional water regulation (7,8). The first identified AQP subtype, AQP1, increases osmotic water permeability by ∼20-fold (9). AQP1 is widely distributed in the ME mucosa and has previously been implicated in the accumulation of effusion in the ME cavity (10).

Glucocorticoids are used clinically as anti-inflammatory drugs to suppress a wide variety of inflammatory and immune responses, although their use as a medical treatment for OME is controversial. Glucocorticoids have been shown to regulate the water balance in several tissues and organs, including the lung, peritoneum and inner ear (11–13). Consequently, an understanding of the effects of glucocorticoids on AQP1 in the ME cavity may provide new insights into the molecular mechanisms involved in transcellular water transport in OME.

In this study, we investigated the expression pattern of aquaporin 1 (AQP1) in a guinea pig model of OME, induced by reversible ET obstruction, in order to analyze the effect of glucocorticoids on AQP1.

## Materials and methods

### Animals and surgery

Male guinea pigs (n=26) weighing between 300 and 450 g were used in this study. Their ears were examined by otomicroscopy and tympanometry to document that the ME was disease-free bilaterally. Then, left ET obstruction was created surgically in all animals. All animals were handled according to the guidelines of the Animal Care and Use Committee of the Affiliated Drum Tower Hospital of Nanjing University School of Medicine (Nanjing, China). The guinea pigs were anesthetized by intraperitoneal injection of a mixture of ketamine (50 mg/kg) and diazepam (5 mg/kg). To create the reversible OME model, the animal’s mouth was propped open and the nasal orifice of the left ET was approached via a transpalatal incision and obstructed with polyvinyl acetal material. The right ear was used as a control. Following surgery, all ears were evaluated daily by otomicroscopy. After effusion was observed in 22 of the animals, two animals were sacrificed for immunohistochemical examination. The others were divided randomly into the OME and dexamethasone (dexa) groups. The dexa group received dexa (5 mg/kg/day) via intraperitoneal injection for 7 days starting on the seventh postoperative day. All animals were sacrificed under deep anesthesia on the fourteenth postoperative day and the left and right temporal bones were removed rapidly for the subsequent experiments.

### Immunohistochemistry

Two guinea pigs with OME were anesthetized as described above and perfused transcardially with physiological saline and 4% paraformaldehyde to fix the tissues *in situ*. The bullae were harvested, fixed with 4% paraformaldehyde at 4°C for 24 h and decalcified with a 10% ethylenediamine tetraacetic acid (EDTA) solution (pH 7.0) at 4°C for 14–17 days. The EDTA solution was changed every day. The specimens were then dehydrated and embedded in paraffin. The tissues were cut into 6-*μ*m sections, deparaffinized and hydrated in phosphate-buffered saline (PBS, pH 7.4). The sections were treated with 3% H_2_O_2_ at room temperature for 30 min to block the endogenous peroxidase activity and then incubated with a rabbit polyclonal antibody against AQP1 (Abcam, Cambridge, MA, USA; 1:400 dilution) at 4°C for 18 h. The sections were then washed with PBS and incubated with a biotin-conjugated secondary antibody against rabbit IgG (PowerVision™ Two-Step Histostaining Reagent; Zhongshan Golden Bridge Biotechnology Co., Ltd., Beijing, China) at room temperature for 30 min. Finally, the sections were incubated with 0.05% 3,3′-diaminobenzidine and then counterstained with Mayer’s hematoxylin. Negative control staining was performed using 0.01 M PBS in place of the primary antibody.

### Protein extraction and western blotting

Protein extraction and western blotting were performed as previously described (10). The ME membranes of the OME and dexa group animals were microdissected and rapidly immersed and homogenized in cold buffer [50 mM Tris-HCl, 0.1% sodium dodecyl sulfate (SDS), 1.0 mM EDTA, 150 mM sodium chloride, 1% Triton X-100, 1% sodium deoxycholate and 1 mM phenylmethylsulfonyl fluoride (PMSF)]. The homogenates were then centrifuged at 12,000 × g at 4°C for 5 min. The supernatants were transferred to new tubes and normalized to a protein content of 5 mg/ml using the Bradford assay; each sample thus prepared was treated with 10X sample buffer and electrophoresed on 10% SDS-polyacrylamide gels. The proteins were transferred to a polyvinyldifluoride membrane, which was incubated first with an affinity-purified polyclonal antibody against AQP1 (rabbit anti-AQP1 affinity-purified polyclonal antibody, 1 mg/ml; species reactivity, rat; Abcam) and then with horseradish peroxidase-conjugated goat anti-rabbit IgG (Santa Cruz Biotechnology, Santa Cruz, CA, USA). The resulting bands were visualized using an enhanced chemiluminescence (ECL) system (Amersham Pharmacia Biotech, Little Chalfont, UK). The labeling density was quantified using ImageQuant software (LabWorks, Portland, OR, USA). The optical densities of the bands from the two groups relative to the densities of the β-actin bands from the same samples were calculated to represent the relative protein abundance.

### Statistical analysis

Association analysis was performed using SPSS software (version 17.0; SPSS, Inc., Chicago, IL, USA). The protein expression of AQP1 was compared between the two groups using the unpaired Student’s t-test with equal variance. Data are expressed as the mean ± standard error. P<0.05 was considered to indicate a statistically significant difference.

## Results

### Animals

**Figure 1:**
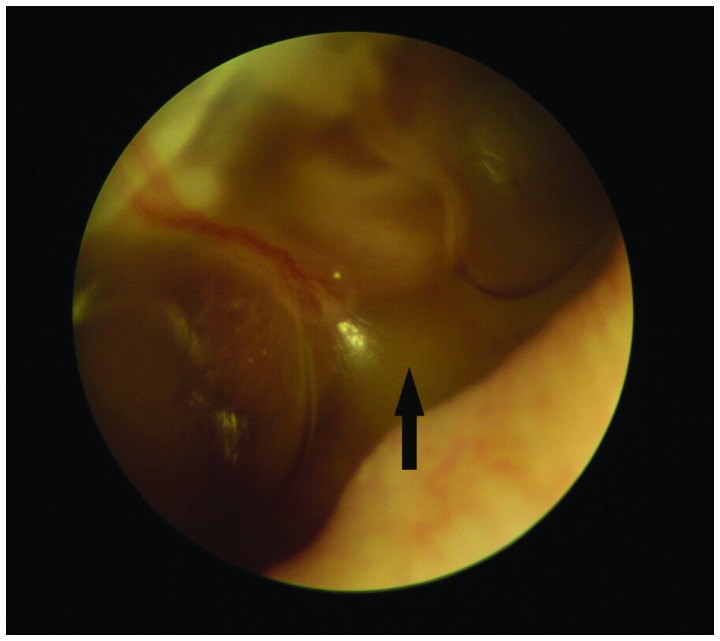
Effusion sign of the tympanic membrane (left ear).

Of the 26 guinea pigs, one died due to systemic failure on the fifth postoperative day and another was observed to have purulent otorrhea in the left ear on the eighth postoperative day. These animals were excluded from further analysis. Of the 24 remaining animals, 22 exhibited effusion 3–7 days after surgery, as indicated by visualization of an air-fluid interface or air bubbles through the tympanic membrane (Fig. 1). None of the control ears exhibited evidence of disease by otomicroscopic examination.

**Figure 2:**
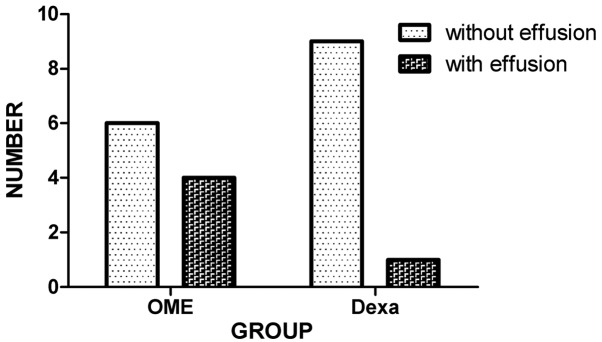
Outcomes of the otitis media with effusion (OME) and dexamethasone (dexa) groups on postoperative day 14.

As shown in Fig. 2, 6 (60%) animals in the OME group and 9 (90%) in the dexa group presented no sign of effusion within the ME cavity on postoperative day 14. These observations were confirmed by microscopic examination of the open bullae.

### Immunohistochemistry

**Figure 3:**
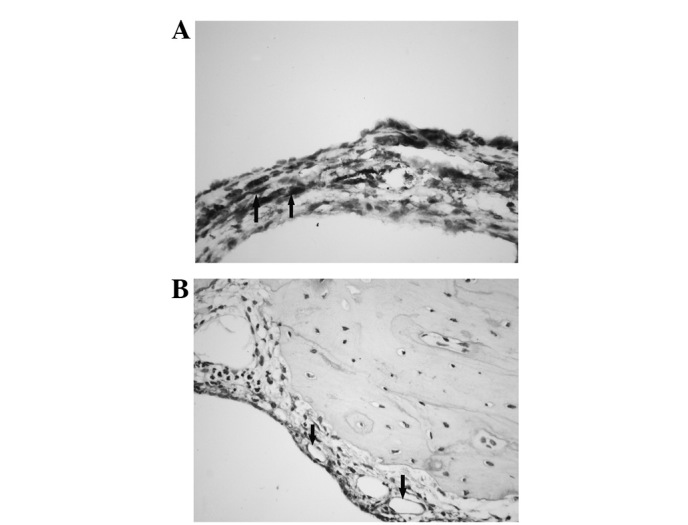
Immunocytochemical localization of aquaporin-1 (AQP1) in the middle ears of guinea pigs with ototis media with effusion (OME). The arrows indicate immunoreactivity in (A) subepithelial fibroblasts and (B) capillary endothelial cells.

Immunohistochemistry was used to determine the cellular distribution of AQP1 in the MEs of guinea pigs with OME. AQP1 expression was observed in the subepithelial fibroblasts and capillary endothelial cells; however, it was not identified in the ciliated and secretory cells within the ME (Fig. 3).

### Western blot analysis

**Figure 4:**
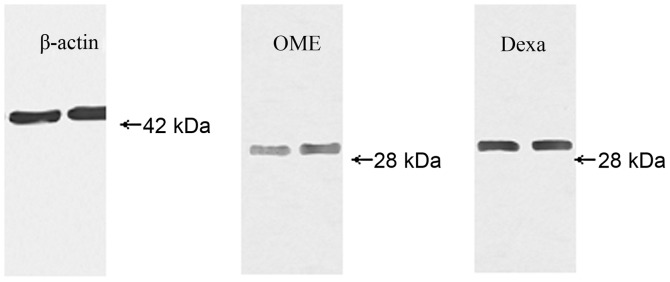
Western blotting for aquaporin-1 (AQP1) in the middle ear mucosa. OME, otitis media with effusion; dexa, dexamethasone.

AQP1 was detected as a 28-kDa protein in the ME mucosa of the animals from the OME and dexa groups (Fig. 4). The level of AQP1 protein was markedly higher in the dexa group than in the OME group (t=2.733, P<0.05; Table I).

## Discussion

The etiology of OME is multifactorial; however, dysfunction of the ET is one of the factors essential to the formation of ME effusion (14). Moreover, inflammation of the ME is often self-limiting and ∼90% of acute episodes (either symptomatic or asymptomatic) resolve within 3 months of presentation with or without medical treatment (15). To date, multiple animal models of OME induced via ET obstruction have been established and used to explore the pathogenesis and treatment of this condition. In the present study, we introduce a novel OME model with reversible ET obstruction obtained via an improved surgical procedure. We blocked the ET with polyvinyl acetal material rather than argent nitrate solution, which produces permanent obstruction (10). As a result, the effusion formed in the ME may be gradually absorbed or pumped out within 2–3 weeks, which is similar to the spontaneous resolution of the majority of cases of OME.

AQP1 is not only the first-identified subtype of the AQP family, but also the most extensively distributed and important. In the plasma membrane, AQP1 molecules form homotetramers, with each 28-kD subunit containing an independent water pore. The atomic-level structure of AQP1 explains its selectivity for water and its ability to facilitate the rapid transport of water across membranes (16). AQP1 is distributed mainly in the endothelial layer of the vasculature throughout the body and helps to increase vasopermeability by facilitating transcellular water movement in the direction of the osmotic gradient (17).

In the present study, we revealed that AQP1 is localized to the cell surface of capillary endothelial cells and fibroblasts in the ME of guinea pigs. Previous findings support the hypothesis that AQP1 is involved in maintaining the ion gradient in the subepithelial milieu of the guinea pig ME (10). Furthermore, we identified that dexa treatment affects AQP1 in a guinea pig model of OME. We administered dexa at a dose of 5 mg/kg since the guinea pig is relatively resistant to glucocorticoids compared with other species, including rats and mice (18). Immunoblotting revealed that dexa treatment increases the expression of AQP1 in the OME-affected ME. In addition, the outcome on postoperative day 14 tended to be better for the dexa group than for the OME group.

Patients suffering from diseases involving edema, including OME, have been successfully treated by the local and oral administration of glucocorticoids. Increasing evidence indicates that in addition to increasing Na^+^/K^+^-ATPase activity (19), glucocorticoids may also regulate water transport in various organs by altering the expression of AQP1. Fukushima *et al* reported that intratympanic injection of steroids upregulates AQP1 mRNA expression in the rat cochlea in a dose-dependent manner (13). Stoenoiu *et al* demonstrated that dexa injection induces the expression of AQP1 in the capillary endothelium of the peritoneal membrane and that a glucocorticoid receptor antagonist inhibits this effect (12). A previous study demonstrated that dexa alleviates pulmonary edema in mice by upregulating the expression of AQP1 in the lung (20). These studies are consistent with our data and indicate that AQP1 is directly involved in these diseases and relieves their symptoms by facilitating water transport. The effect of glucocorticoids on AQP1 may be due to the presence of the multiple glucocorticoid response elements that have been identified in the promoters of mammalian AQP1 genes (21,22). This regulatory mechanism warrants further investigation.

One limitation of the present study is that only a single dose and duration of dexa treatment was used. Further studies with a greater number of groups and analysis of the dose- and time-dependence of the response should be performed to elucidate the effect of dexa on AQP1 more fully.

In conclusion, corticosteroids induce the expression of AQP1 protein in the mucosa of the ME. Our data emphasize the significance of AQP1 in the pathophysiology of OME and suggest that glucocorticoids regulate water homeostasis via an AQP1 pathway, which may be a new target for drug therapy.

## Figures and Tables

**Table I:** Expression of AQP1 protein in the two groups.

Group	N	Protein amount
OME	10	0.2435±0.04422
Dexa	10	0.4145±0.04427^a^

Data are presented as mean ± standard error.

a P<0.05, compared with the OME group. AQP1, aquaporin-1; OME, otitis media with effusion; dexa, dexamethasone.

## References

[1] Morris LM, DeGagne JM, Kempton JB, Hausman F, Trune DR (2012). Mouse middle ear ion homeostasis channels and intercellular junctions. PLoS One.

[2] Topcuoglu N, Keskin F, Ciftci S, Paltura C, Kulekci M, Ustek D, Kulekci G (2012). Relationship between oral anaerobic bacteria and otitis media with effusion. Int J Med Sci.

[3] Bluestone CD, Klein JO (2007). Physiology, pathophysiology and pathogenesis. Otitis Media in Infants and Children.

[4] Post JC (2001). Direct evidence of bacterial biofilms in otitis media. Laryngoscope.

[5] Darrow DH, Dash N, Derkay CS (2003). Otitis media: concepts and controversies. Curr Opin Otolaryngol Head Neck Surg.

[6] Takata K, Matsuzaki T, Tajika Y (2004). Aquaporins: water channel proteins of the cell membrane. Prog Histochem Cytochem.

[7] Altuntas A, Yilmaz MD, Aktepe F, Kahveci OK, Derekoy S, Dilek H, Serteser M (2006). Expression and distribution of aquaporin-1 in nasal polyps: does it have any significance in edema formation?. Am J Rhinol.

[8] Takeda T, Taguchi D (2009). Aquaporins as potential drug targets for Meniere’s disease and its related diseases. Handb Exp Pharmacol.

[9] Hasegawa H, Ma T, Skach W, Matthay MA, Verkman AS (1994). Molecular cloning of a mercurial-insensitive water channel expressed in selected water-transporting tissues. J Biol Chem.

[10] Zhang Q, Liu C, Gao X, Hu Y, Guo W, Sun J, Li X (2009). Expression pattern of aquaporin 1 in the middle ear of the guinea pig with secretory otitis media. ORL J Otorhinolaryngol Relat Spec.

[11] Liu H, Hooper SB, Armugam A, Dawson N, Ferraro T, Jeyaseelan K, Thiel A, Koukoulas I, Wintour EM (2003). Aquaporin gene expression and regulation in the ovine fetal lung. J Physiol.

[12] Stoenoiu MS, Ni J, Verkaeren C, Debaix H, Jonas JC, Lameire N, Verbavatz JM, Devuyst O (2003). Corticosteroids induce expression of aquaporin-1 and increase transcellular water transport in rat peritoneum. J Am Soc Nephrol.

[13] Fukushima M, Kitahara T, Uno Y, Fuse Y, Doi K, Kubo T (2002). Effects of intratympanic injection of steroids on changes in the rat inner ear aquaporin expression. Acta Otolaryngol.

[14] Honjo I, Okazaki N, Nozoe T, Ushiro K, Kumazawa T (1981). Experimental study of the pumping function of the Eustachian tube. Acta Otolaryngol (Stockh).

[15] Hebda PA, Piltcher OB, Swarts JD, Alper CM, Zeevi A, Doyle WJ (2002). Cytokine profiles in a rat model of otitis media with effusion caused by eustachian tube obstruction with and without *Streptococcus pneumoniae* infection. Laryngoscope.

[16] Kozono D, Yasui M, King LS, Agre P (2002). Aquaporin water channels: Atomic structure molecular dynamics meet clinical medicine. J Clin Invest.

[17] Verkman AS (2002). Aquaporin water channels and endothelial cell function. J Anat.

[18] Nagata T, Nabe T, Fujii M, Mizutani N, Kohno S (2008). Effects of multiple dexamethasone treatments on aggravation of allergic conjunctivitis associated with mast cell hyperplasia. Biol Pharm Bull.

[19] Kim CR, Sadowska GB, Newton SA, Merino M, Petersson KH, Padbury JF, Stonestreet BS (2011). Na^+^,K^+^-ATPase activity and subunit protein expression: ontogeny and effects of exogenous and endogenous steroids on the cerebral cortex and renal cortex of sheep. Reprod Sci.

[20] Dong C, Wang G, Li B, Xiao K, Ma Z, Huang H, Wang X, Bai C (2012). Anti-asthmatic agents alleviate pulmonary edema by upregulating AQP1 and AQP5 expression in the lungs of mice with OVA-induced asthma. Respir Physiol Neurobiol.

[21] de Arteaga J, Ledesma F, Garay G, Chiurchiu C, de la Fuente J, Douthat W, Massari P, Terryn S, Devuyst O (2011). High-dose steroid treatment increases free water transport in peritoneal dialysis patients. Nephrol Dial Transplant.

[22] Moon C, King LS, Agre P (1997). Aqp1 expression in erythroleukemia cells: genetic regulation of glucocorticoid and chemical induction. Am J Physiol.

